# 7-Chloro-6,8-dinitro­quinazolin-4(3*H*)-one acetic acid monosolvate

**DOI:** 10.1107/S1600536811055735

**Published:** 2012-01-07

**Authors:** Yongqiang Yu

**Affiliations:** a Department of Organic Chemistry, China Pharmaceutical University, Nanjing 210009, People’s Republic of China

## Abstract

In the title compound, C_8_H_3_ClN_4_O_5_·C_2_H_4_O_2_, both the nitro groups are close to perpendicular [dihedral angles = 67.62 (15) and 86.73 (12)°] to the almost planar quinazoline unit [r.m.s. deviation = 0.014Å]. In the crystal, both the quinazoline and acetic acid mol­ecules form inversion dimers linked by pairs of N—H⋯O and O—H⋯O hydrogen bonds, respectively. *R*_2_^2^(8) loops arise in each case.

## Related literature

For background to the biological properties of quinazoline derivatives, see: Pandeya *et al.* (1999 ▶); Tereshima *et al.* (1995 ▶); Wolfe *et al.* (1990 ▶). For a related structure, see: Srinivasan *et al.* (2011 ▶). For graph-set notation, see: Bernstein *et al.* (1995 ▶).
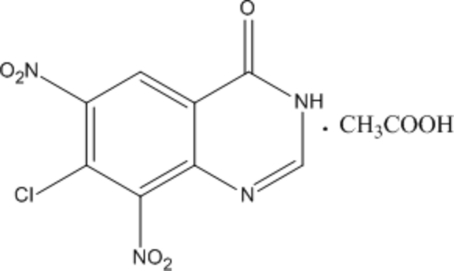


## Experimental

### 

#### Crystal data

C_8_H_3_ClN_4_O_5_·C_2_H_4_O_2_
*M* *_r_* = 330.65
Triclinic, 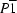

*a* = 7.3041 (12) Å
*b* = 9.3952 (16) Å
*c* = 9.6850 (16) Å
α = 83.813 (2)°
β = 88.172 (2)°
γ = 89.033 (2)°
*V* = 660.35 (19) Å^3^
*Z* = 2
Mo *K*α radiation
μ = 0.33 mm^−1^
*T* = 293 K
0.25 × 0.23 × 0.21 mm

#### Data collection

Rigaku SCXmini diffractometer
4758 measured reflections
2332 independent reflections
1810 reflections with *I* > 2σ(*I*)
*R*_int_ = 0.023

#### Refinement

*R*[*F* ^2^ > 2σ(*F* ^2^)] = 0.045
*wR*(*F* ^2^) = 0.123
*S* = 1.05
2332 reflections
200 parameters
H-atom parameters constrained
Δρ_max_ = 0.34 e Å^−3^
Δρ_min_ = −0.32 e Å^−3^

### 

Data collection: *CrystalClear* (Rigaku, 2005 ▶); cell refinement: *CrystalClear*; data reduction: *CrystalClear*; program(s) used to solve structure: *SHELXS97* (Sheldrick, 2008 ▶); program(s) used to refine structure: *SHELXL97* (Sheldrick, 2008 ▶); molecular graphics: *SHELXTL* (Sheldrick, 2008 ▶); software used to prepare material for publication: *SHELXTL*.

## Supplementary Material

Crystal structure: contains datablock(s) I, global. DOI: 10.1107/S1600536811055735/hb6577sup1.cif

Supplementary material file. DOI: 10.1107/S1600536811055735/hb6577Isup2.cdx

Structure factors: contains datablock(s) I. DOI: 10.1107/S1600536811055735/hb6577Isup3.hkl

Supplementary material file. DOI: 10.1107/S1600536811055735/hb6577Isup4.cml

Additional supplementary materials:  crystallographic information; 3D view; checkCIF report

## Figures and Tables

**Table 1:** Hydrogen-bond geometry (Å, °)

*D*—H⋯*A*	*D*—H	H⋯*A*	*D*⋯*A*	*D*—H⋯*A*
N4—H4*A*⋯O1^i^	0.86	1.97	2.827 (3)	173
O6—H100⋯O7^ii^	0.83	1.86	2.665 (3)	163

Symmetry codes: (i) 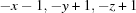; (ii) 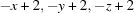.

**Fig. 1.**
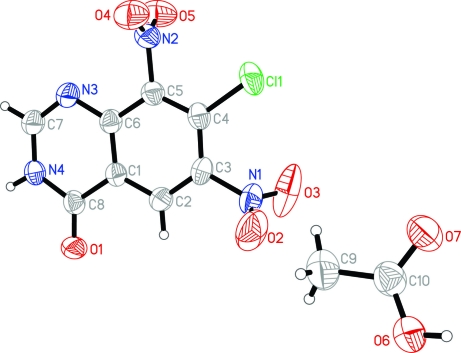
The molecular structure of the title compound with displacement ellipsoids drawn at the 50% probability level.

**Fig. 2.**
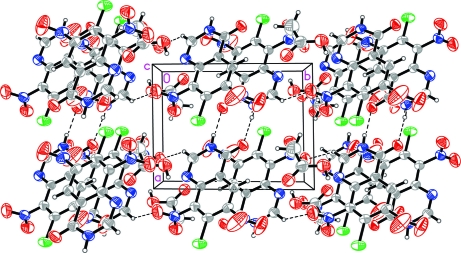
Partial packing view of the title compound, viewed down the c axis.

## supplementary crystallographic information

### Experimental

7-Chloro-quinazolin-4(3*H*)-one(18.0 g, 100 mmol) was added portionwise to a stirred mixture of concentrated sulfuric acid (60 ml) and fuming nitric acid (60 ml) which had been cooled to 273 K. The mixture was stirred at ambient temperature for 1 h and then heated to 373 K for 4 h. Then it was poured into 800 g crush ice. The precipitate was isolated, washed with water and dried. Recrystallization from acetic acid gives 7-chloro-6,8-dinitroquinazolin-4(3*H*)-one (14.1 g, 52.1%). Yellow blocks were obtained by slow evaporation of an acetic acid solution at room temperature. m.p.:573 K (decomp.) ^1^H-NMR (DMSO– d_6_, δ (p.p.m.)): 13.2 (1*H*, brs), 8.89 (1*H*, s), 8.44 (1*H*, s), CI—MS (m/e): 271.5 (*M*+1).

### Refinement

H atoms bonded to C and N atoms were placed in calculated positions (C—H = 0.93–0.96 Å and N—H = 0.86 Å) and included in the riding model approximation. For all H atoms *U*iso (H) = 1.2*U*iso (C, *N*) or 1.5*U*iso (C).

### Crystal data

**Table:**

C_8_H_3_ClN_4_O_5_·C_2_H_4_O_2_	*Z* = 2
*M**_r_* = 330.65	*F*(000) = 336
Triclinic, *P*1	*D*_x_ = 1.663 Mg m^−^^3^
Hall symbol: -P 1	Mo *K*α radiation, λ = 0.71073 Å
*a* = 7.3041 (12) Å	Cell parameters from 30 reflections
*b* = 9.3952 (16) Å	θ = 3–25°
*c* = 9.6850 (16) Å	µ = 0.33 mm^−^^1^
α = 83.813 (2)°	*T* = 293 K
β = 88.172 (2)°	Block, yellow
γ = 89.033 (2)°	0.25 × 0.23 × 0.21 mm
*V* = 660.35 (19) Å^3^	

### Data collection

**Table:**

Rigaku SCXmini diffractometer	*R*_int_ = 0.023
Radiation source: fine-focus sealed tube	θ_max_ = 25.1°, θ_min_ = 2.1°
graphite	*h* = −8→8
ω scans	*k* = −11→11
4758 measured reflections	*l* = −11→11
2332 independent reflections	3 standard reflections every 150 reflections
1810 reflections with *I* > 2σ(*I*)	intensity decay: none

### Refinement

**Table:**

Refinement on *F*^2^	Primary atom site location: structure-invariant direct methods
Least-squares matrix: full	Secondary atom site location: difference Fourier map
*R*[*F*^2^ > 2σ(*F*^2^)] = 0.045	Hydrogen site location: inferred from neighbouring sites
*wR*(*F*^2^) = 0.123	H-atom parameters constrained
*S* = 1.05	*w* = 1/[σ^2^(*F*_o_^2^) + (0.0589*P*)^2^ + 0.2567*P*] where *P* = (*F*_o_^2^ + 2*F*_c_^2^)/3
2332 reflections	(Δ/σ)_max_ < 0.001
200 parameters	Δρ_max_ = 0.34 e Å^−^^3^
0 restraints	Δρ_min_ = −0.32 e Å^−^^3^

### Special details

**Table:**

Geometry. All e.s.d.'s (except the e.s.d. in the dihedral angle between two l.s. planes) are estimated using the full covariance matrix. The cell e.s.d.'s are taken into account individually in the estimation of e.s.d.'s in distances, angles and torsion angles; correlations between e.s.d.'s in cell parameters are only used when they are defined by crystal symmetry. An approximate (isotropic) treatment of cell e.s.d.'s is used for estimating e.s.d.'s involving l.s. planes.
Refinement. Refinement of *F*^2^ against ALL reflections. The weighted *R*-factor *wR* and goodness of fit *S* are based on *F*^2^, conventional *R*-factors *R* are based on *F*, with *F* set to zero for negative *F*^2^. The threshold expression of *F*^2^ > σ(*F*^2^) is used only for calculating *R*-factors(gt) *etc*. and is not relevant to the choice of reflections for refinement. *R*-factors based on *F*^2^ are statistically about twice as large as those based on *F*, and *R*- factors based on ALL data will be even larger.

### Fractional atomic coordinates and isotropic or equivalent isotropic displacement parameters (Å^2^)

**Table:**

	*x*	*y*	*z*	*U*_iso_*/*U*_eq_	
Cl1	0.45451 (9)	0.28014 (8)	0.85154 (7)	0.0573 (3)	
C1	−0.0819 (3)	0.4157 (2)	0.6577 (2)	0.0366 (5)	
C2	0.0022 (3)	0.5048 (3)	0.7415 (3)	0.0410 (6)	
H2	−0.0516	0.5922	0.7573	0.049*	
C3	0.1646 (3)	0.4627 (3)	0.8006 (3)	0.0417 (6)	
C4	0.2506 (3)	0.3322 (3)	0.7788 (2)	0.0406 (6)	
C5	0.1668 (3)	0.2480 (2)	0.6936 (3)	0.0400 (6)	
C6	−0.0007 (3)	0.2851 (2)	0.6312 (2)	0.0386 (6)	
C7	−0.2288 (3)	0.2322 (3)	0.4948 (3)	0.0474 (6)	
H7	−0.2823	0.1714	0.4385	0.057*	
C8	−0.2573 (3)	0.4555 (3)	0.5944 (2)	0.0399 (6)	
C9	0.6882 (5)	0.8879 (4)	0.7816 (4)	0.0864 (11)	
H9A	0.7294	0.9190	0.6883	0.130*	
H9B	0.5696	0.9291	0.7990	0.130*	
H9C	0.6807	0.7854	0.7936	0.130*	
C10	0.8197 (4)	0.9348 (3)	0.8806 (3)	0.0577 (7)	
N1	0.2488 (3)	0.5589 (3)	0.8899 (3)	0.0516 (6)	
N2	0.2536 (3)	0.1115 (2)	0.6652 (2)	0.0469 (5)	
N3	−0.0740 (3)	0.1927 (2)	0.5468 (2)	0.0473 (5)	
N4	−0.3205 (3)	0.3552 (2)	0.5155 (2)	0.0424 (5)	
H4A	−0.4242	0.3712	0.4768	0.051*	
O1	−0.3420 (2)	0.56686 (18)	0.6085 (2)	0.0546 (5)	
O2	0.3030 (4)	0.6707 (3)	0.8373 (3)	0.0982 (9)	
O3	0.2565 (4)	0.5191 (4)	1.0109 (3)	0.1116 (11)	
O4	0.3643 (3)	0.1136 (2)	0.5693 (2)	0.0664 (6)	
O5	0.2093 (3)	0.0052 (2)	0.7389 (2)	0.0735 (6)	
O6	0.8157 (3)	1.0667 (2)	0.8998 (2)	0.0722 (6)	
H100	0.9096	1.0894	0.9368	0.087*	
O7	0.9282 (3)	0.8452 (2)	0.9401 (2)	0.0691 (6)	

### Atomic displacement parameters (Å^2^)

**Table:**

	*U*^11^	*U*^22^	*U*^33^	*U*^12^	*U*^13^	*U*^23^
Cl1	0.0381 (4)	0.0782 (5)	0.0559 (5)	0.0006 (3)	−0.0151 (3)	−0.0054 (3)
C1	0.0351 (12)	0.0392 (12)	0.0357 (13)	−0.0021 (10)	−0.0042 (10)	−0.0032 (10)
C2	0.0410 (14)	0.0386 (12)	0.0440 (14)	−0.0034 (10)	−0.0038 (11)	−0.0063 (10)
C3	0.0399 (13)	0.0480 (14)	0.0387 (13)	−0.0109 (11)	−0.0032 (11)	−0.0097 (11)
C4	0.0329 (13)	0.0504 (14)	0.0383 (13)	−0.0033 (11)	−0.0033 (11)	−0.0023 (11)
C5	0.0368 (13)	0.0403 (13)	0.0431 (14)	−0.0001 (10)	−0.0039 (11)	−0.0049 (11)
C6	0.0369 (13)	0.0394 (12)	0.0398 (13)	−0.0043 (10)	−0.0034 (11)	−0.0049 (10)
C7	0.0445 (15)	0.0473 (14)	0.0529 (16)	−0.0007 (12)	−0.0117 (13)	−0.0140 (12)
C8	0.0369 (13)	0.0418 (13)	0.0412 (14)	−0.0029 (11)	−0.0079 (11)	−0.0030 (11)
C9	0.092 (3)	0.080 (2)	0.090 (3)	−0.004 (2)	−0.037 (2)	−0.009 (2)
C10	0.0504 (17)	0.0579 (18)	0.0637 (19)	−0.0042 (14)	−0.0068 (14)	0.0007 (14)
N1	0.0443 (13)	0.0635 (15)	0.0504 (15)	−0.0095 (11)	−0.0075 (11)	−0.0179 (12)
N2	0.0406 (12)	0.0462 (13)	0.0546 (14)	0.0049 (10)	−0.0073 (11)	−0.0084 (11)
N3	0.0451 (12)	0.0459 (12)	0.0537 (13)	−0.0001 (10)	−0.0124 (10)	−0.0154 (10)
N4	0.0346 (11)	0.0472 (11)	0.0470 (12)	0.0002 (9)	−0.0135 (9)	−0.0090 (9)
O1	0.0483 (11)	0.0442 (10)	0.0745 (13)	0.0110 (8)	−0.0226 (10)	−0.0169 (9)
O2	0.132 (2)	0.0697 (15)	0.0967 (19)	−0.0419 (15)	−0.0373 (17)	−0.0089 (14)
O3	0.135 (3)	0.154 (3)	0.0519 (15)	−0.075 (2)	−0.0054 (16)	−0.0282 (16)
O4	0.0610 (13)	0.0697 (13)	0.0686 (14)	0.0091 (10)	0.0139 (11)	−0.0138 (11)
O5	0.0827 (16)	0.0466 (11)	0.0875 (16)	0.0069 (10)	0.0103 (13)	0.0048 (11)
O6	0.0634 (13)	0.0667 (14)	0.0885 (16)	0.0045 (10)	−0.0276 (12)	−0.0107 (12)
O7	0.0586 (12)	0.0571 (12)	0.0892 (16)	−0.0049 (10)	−0.0190 (12)	0.0094 (11)

### Geometric parameters (Å, °)

**Table:**

Cl1—C4	1.710 (2)	C8—O1	1.225 (3)
C1—C2	1.390 (3)	C8—N4	1.371 (3)
C1—C6	1.399 (3)	C9—C10	1.482 (4)
C1—C8	1.463 (3)	C9—H9A	0.9600
C2—C3	1.368 (3)	C9—H9B	0.9600
C2—H2	0.9300	C9—H9C	0.9600
C3—C4	1.402 (3)	C10—O7	1.253 (4)
C3—N1	1.471 (3)	C10—O6	1.273 (3)
C4—C5	1.367 (3)	N1—O2	1.186 (3)
C5—C6	1.404 (3)	N1—O3	1.193 (3)
C5—N2	1.470 (3)	N2—O5	1.206 (3)
C6—N3	1.380 (3)	N2—O4	1.210 (3)
C7—N3	1.285 (3)	N4—H4A	0.8600
C7—N4	1.356 (3)	O6—H100	0.8254
C7—H7	0.9300		
			
C2—C1—C6	120.8 (2)	O1—C8—C1	124.6 (2)
C2—C1—C8	121.1 (2)	N4—C8—C1	113.4 (2)
C6—C1—C8	118.0 (2)	C10—C9—H9A	109.5
C3—C2—C1	119.4 (2)	C10—C9—H9B	109.5
C3—C2—H2	120.3	H9A—C9—H9B	109.5
C1—C2—H2	120.3	C10—C9—H9C	109.5
C2—C3—C4	122.0 (2)	H9A—C9—H9C	109.5
C2—C3—N1	118.0 (2)	H9B—C9—H9C	109.5
C4—C3—N1	120.0 (2)	O7—C10—O6	123.4 (3)
C5—C4—C3	117.3 (2)	O7—C10—C9	119.6 (3)
C5—C4—Cl1	120.62 (19)	O6—C10—C9	117.0 (3)
C3—C4—Cl1	122.05 (19)	O2—N1—O3	124.5 (3)
C4—C5—C6	123.1 (2)	O2—N1—C3	117.9 (2)
C4—C5—N2	119.3 (2)	O3—N1—C3	117.5 (3)
C6—C5—N2	117.6 (2)	O5—N2—O4	124.6 (2)
N3—C6—C1	124.0 (2)	O5—N2—C5	117.7 (2)
N3—C6—C5	118.7 (2)	O4—N2—C5	117.7 (2)
C1—C6—C5	117.3 (2)	C7—N3—C6	115.4 (2)
N3—C7—N4	125.3 (2)	C7—N4—C8	123.8 (2)
N3—C7—H7	117.3	C7—N4—H4A	118.1
N4—C7—H7	117.3	C8—N4—H4A	118.1
O1—C8—N4	122.0 (2)	C10—O6—H100	111.3

### Hydrogen-bond geometry (Å, °)

**Table:**

*D*—H···*A*	*D*—H	H···*A*	*D*···*A*	*D*—H···*A*
N4—H4A···O1^i^	0.86	1.97	2.827 (3)	173
O6—H100···O7^ii^	0.83	1.86	2.665 (3)	163

Symmetry codes: (i) −*x*−1, −*y*+1, −*z*+1; (ii) −*x*+2, −*y*+2, −*z*+2.
